# Integration of VDR genome wide binding and GWAS genetic variation data reveals co-occurrence of VDR and NF-κB binding that is linked to immune phenotypes

**DOI:** 10.1186/s12864-017-3481-4

**Published:** 2017-02-06

**Authors:** Prashant K. Singh, Patrick R. van den Berg, Mark D. Long, Angie Vreugdenhil, Laurie Grieshober, Heather M. Ochs-Balcom, Jianmin Wang, Sylvie Delcambre, Sami Heikkinen, Carsten Carlberg, Moray J. Campbell, Lara E. Sucheston-Campbell

**Affiliations:** 1 Department of Pharmacology & Therapeutics, Roswell Park Cancer Institute, Buffalo, NY 14263 USA; 2 Leiden institute of Physics, Leiden University, 2300 RA Leiden, Netherlands; 3 Department of Epidemiology and Environmental Health, School of Public Health and Health Professions, University at Buffalo, Buffalo, NY 14214 USA; 4 Department of Biostatistics and Bioinformatics, Roswell Park Cancer Institute, Buffalo, NY 14263 USA; 5 Luxembourg Centre for Systems Biomedicine, 6 Avenue du Swing, 4367 Belvaux, Luxembourg; 6 School of Medicine, Institute of Biomedicine, University of Eastern Finland, Kuopio, 70211 Finland; 7 Division of Pharmaceutics and Pharmaceutical Chemistry, College of Pharmacy, 536 Parks Hall, The Ohio State University, Columbus, OH 43210 USA; 8 Division of Pharmacy Practice and Science, College of Pharmacy, 604 Riffe Building, The Ohio State University, Columbus, OH 43210 USA; 9 Department of Veterinary Biosciences, College of Veterinary Medicine, The Ohio State University, Columbus, OH 43210 USA

**Keywords:** VDR, GWAS, SNP, Immune function, ChIP-seq, NF-κB, Linkage disequilibrium, Nuclear receptor superfamily, Cistrome, DR3 motif

## Abstract

**Background:**

The nuclear hormone receptor superfamily acts as a genomic sensor of diverse signals. Their actions are often intertwined with other transcription factors. Nuclear hormone receptors are targets for many therapeutic drugs, and include the vitamin D receptor (VDR). VDR signaling is pleotropic, being implicated in calcaemic function, antibacterial actions, growth control, immunomodulation and anti-cancer actions. Specifically, we hypothesized that the biologically significant relationships between the VDR transcriptome and phenotype-associated biology could be discovered by integrating the known VDR transcription factor binding sites and all published trait- and disease-associated SNPs. By integrating VDR genome-wide binding data (ChIP-seq) with the National Human Genome Research Institute (NHGRI) GWAS catalog of SNPs we would see where and which target gene interactions and pathways are impacted by inherited genetic variation in VDR binding sites, indicating which of VDR’s multiple functions are most biologically significant.

**Results:**

To examine how genetic variation impacts VDR function we overlapped 23,409 VDR genomic binding peaks from six VDR ChIP-seq datasets with 191,482 SNPs, derived from GWAS-significant SNPs (Lead SNPs) and their correlated variants (*r* ^2^ > 0.8) from HapMap3 and the 1000 genomes project. In total, 574 SNPs (71 Lead and 503 SNPs in linkage disequilibrium with Lead SNPs) were present at VDR binding loci and associated with 211 phenotypes. For each phenotype a hypergeometric test was used to determine if SNPs were enriched at VDR binding sites. Bonferroni correction for multiple testing across the 211 phenotypes yielded 42 SNPs that were either disease- or phenotype-associated with seven predominately immune related including self-reported allergy; esophageal cancer was the only cancer phenotype. Motif analyses revealed that only two of these 42 SNPs reside within a canonical VDR binding site (DR3 motif), and that 1/3 of the 42 SNPs significantly impacted binding and gene regulation by other transcription factors, including NF-κB. This suggests a plausible link for the potential cross-talk between VDR and NF-κB.

**Conclusions:**

These analyses showed that VDR peaks are enriched for SNPs associated with immune phenotypes suggesting that VDR immunomodulatory functions are amongst its most important actions. The enrichment of genetic variation in non-DR3 motifs suggests a significant role for the VDR to bind in multimeric complexes containing other transcription factors that are the primary DNA binding component. Our work provides a framework for the combination of ChIP-seq and GWAS findings to provide insight into the underlying phenotype-associated biology of a given transcription factor.

**Electronic supplementary material:**

The online version of this article (doi:10.1186/s12864-017-3481-4) contains supplementary material, which is available to authorized users.

## Background

The annotation of the human genome by ENCODE, Roadmap Epigenome, FANTOM and other consortia [1–6] has revealed the widespread distribution of transcription factor binding loci throughout the genome. These patterns, known as cistromes, include numerous enhancers that are often extremely distal to genes [7–13]. The diversity and function of these distal enhancer sites suggests a hitherto unsuspected level of complexity to transcriptional control (reviewed in [14]). Recently, it has emerged that transcription factors including nuclear hormone receptors can bind at enhancers in both direct contact with DNA (*cis*) and indirectly in contact with another protein that in turn bind to DNA (*trans*). Furthermore this type of *trans* binding is often absent of canonical motifs but associated with significant levels of transcription factor clustering [15–17]. In parallel, large scale genome-wide association studies (GWAS) of genetic variation have revealed that the vast majority of SNPs are contained in areas of the genome that are outside of gene exons, and therefore do not have the potential to make a direct contribution to protein structure [18]. Taken together, these findings of genomic distribution of transcription factor binding sites and widespread genetic variation at non-coding sites raises the possibility that phenotype- and disease-associated SNPs at distal regions impact transcription factor binding that in turn is associated with disease [18, 19].

Testing the possibility that genetic variation impacts transcription factor function that underpins trait differences and disease phenotypes is analytically challenging, given the size of the datasets and the potential for false discovery. Various groups have addressed this challenge; notably, both the ENCODE and Roadmap Epigenome consortia leveraged the remarkable volume of ChIP-seq xdata they generated and merged the binding sites with GWAS data to reveal and rank sites where SNPs appear to have a significant impact on the activity of multiple transcription factors [4, 20, 21]. Whilst these analyses represented a highly comprehensive approach, undertaking ChIP-seq with several hundred DNA binding factors, this still represents a fraction of the approximately 3000 different transcription regulatory proteins in humans.

Rather than taking a SNP-centric approach [22], we have approached this problem by focusing on a specific transcription factor (one that is outside of those analyzed by the ENCODE and Roadmap Epigenome consortia). This is potentially a complementary and attractive approach, as it overlays with the work flow of wet-lab based biologists who generally approach biological questions through the lens of an in-depth understanding of a single transcription factor, or transcription factor family. Specifically, we hypothesize that the biologically significant relationships between transcription factor genomic interactions and phenotype-associated biology can be discovered by integrating the known binding sites and all trait- and disease-associated common SNPs. This exploits the value of both datasets to identify the biologically significant intersection of transcription factor binding and genetic variation.

In the current study we have addressed the challenge of dissecting the multiple actions transcription factor function by examining nuclear hormone receptor actions. The nuclear hormone receptor superfamily acts as an integrated genomic sensor of dietary, environmental and hormonal signals. These receptors represent some of the most successful examples of targeted therapies [23–26], and in human disease they represent the target for approximately 15% of all pharmacologic drugs [27]. Furthermore members of this superfamily are expressed in virtually all cell types and functionally are highly integrated both with each other [28–30] and with the actions of other signaling pathways [31].

As a model transcription factor we selected the vitamin D receptor (VDR/NR1I1) [32] from this superfamily, and investigated the association between genetic variation at VDR binding sites and disease susceptibility. The VDR is an attractive transcription factor with which to dissect pleotropic functions because its actions have been identified or implicated in many physiological processes. VDR actions include classical endocrine functions to regulate serum calcium levels, and are also related to the control of cell proliferation and differentiation [33], anti-bacterial functions [34–38] and immuno-modulatory functions [39, 40]. As a result, roles for VDR function and dysfunction have been implicated in a wide range of complex phenotypes including autoimmunity, diabetes, cardiac health and cancer [23, 33]. Reflecting the potential importance of this receptor in public health, there are a number of ongoing large-scale prospective studies that aim to address whether supplementation of serum vitamin D levels can have a significant health impact [41–43].

Attempts to explain this have focused heavily on how the VDR impacts gene regulation. At the level of candidate target genes, the VDR has been demonstrated to functionally interact with a wide range of other transcription factors, including SP1 [44, 45] GATA4 [46], HNF4α [47], CTCF [48], FOXO4 [49], STAT3 [50, 51], and NF-κB [52, 53]. This latter interaction with NF-κB is also supported by a number of studies that examined the interactions between VDR signaling, and the control of inflammatory phenotypes, and specifically the cross-talk with NF-κB actions. These interactions are relatively well described in intestinal systems and include direct antagonism between VDR and NF-κB over the controls of shared target genes [54–60].

Efforts to understand the DNA sequences bound by the VDR have built on findings from other nuclear hormone receptors [61–63], and biochemical approaches on candidate target gene promoter regions. These approaches identified a dual hexameric DNA motif spaced by 3 bp, a so-called DR3 motif [64, 65], is bound with high affinity by the VDR. However, other potential motifs have also been identified [66], and the application of ChIP-seq approaches to nuclear hormone receptors has revealed binding site diversity and the importance of flanking regions for cofactors to be biologically important to determine function [67]. For example, VDR ChIP-seq studies have been performed in different human cell types [8–11, 68], in the presence and/or absence of ligand, and revealed the impact of ligand binding on VDR genomic targeting.

Each analysis revealed approximately 2,000 to 6,000 genomic loci normally distributed around transcription start sites (TSS), reflecting the binomial distributions found for other transcription factors [21, 68]. Another important finding from these studies is that DR3 motif appears to be the minority genomic element that directly binds the VDR, and perhaps only 20% of genomic VDR binding sites contain a DR3 motif. This low number may in part reflect algorithm limitations for de novo motif discovery. Although this number is increased following ligand treatment (reviewed in [23]), this nonetheless suggests that the classical DR3 motif is the minority motif bound by the VDR. This also suggests that there is considerable diversity in the DNA sequences bound by VDR complexes, and that VDR participates in both *cis* and *trans* genomic interactions.

The apparently interactive nature of the VDR with other transcription factors and the diversity in observed binding sites for the VDR were catalysts for the current study. Given the multiple actions that the VDR is implicated in and the heterogeneity of VDR genomic binding sites we further hypothesized that genetic variation may be exploited to identify critical VDR genomic interactions.

**Fig. 1:**
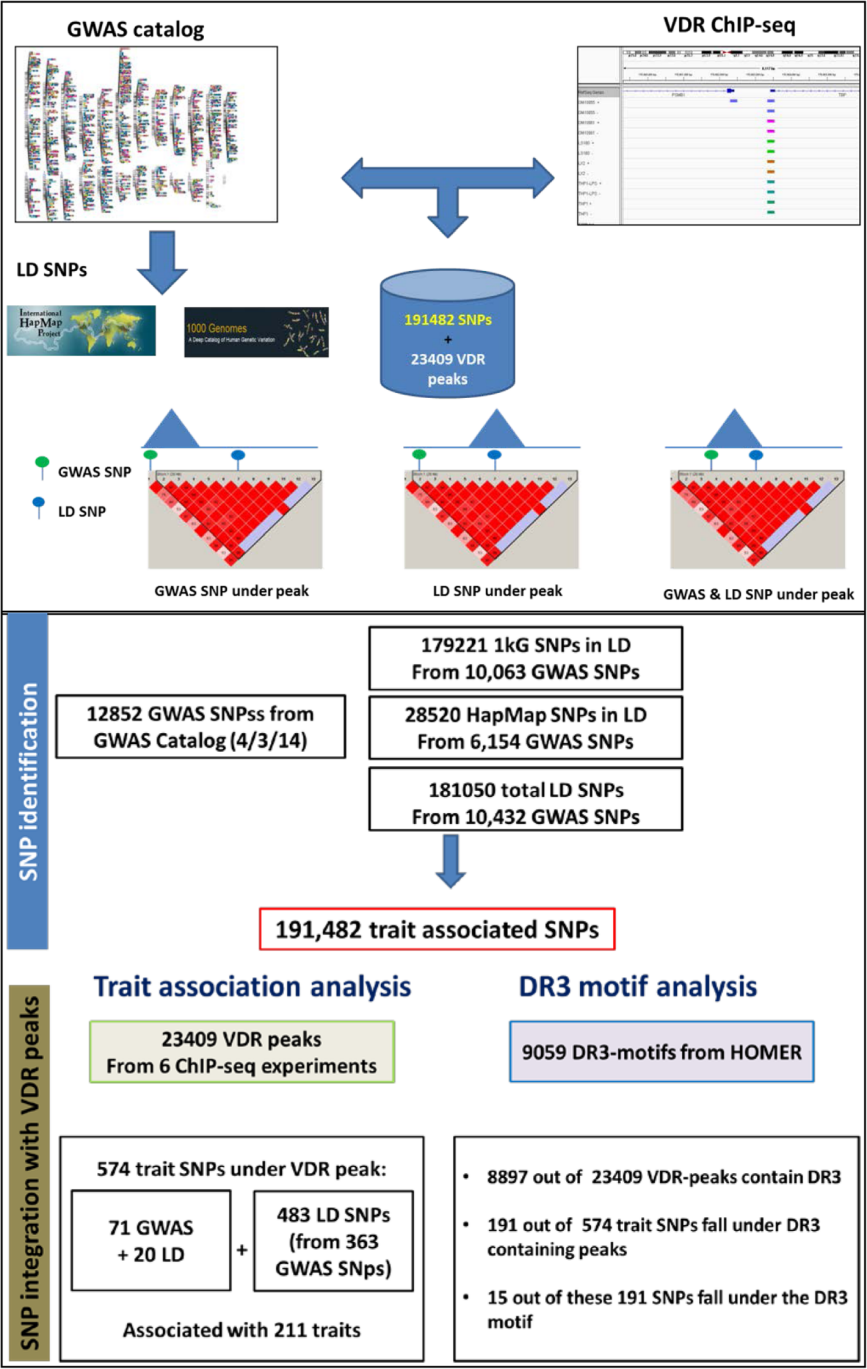
Schematic workflow and key findings of the study. (*Top panel*) shows the SNPs and ChIP-seq datasets. Examples of possible overlap of Lead SNPs and SNPs in LD with Lead SNPs at genomic VDR loci are shown with examples of LD blocks. (*Bottom panel*) shows the number of SNPs identified from individual data sets (GWAS catalog, HapMap3 and 1000 genome project). It also shows the number of SNPs under VDR peaks and SNPs in a DR3-type motif

Therefore the present study aimed to integrate VDR genomic binding data (ChIP-seq) with GWAS SNPs to provide novel insight into the interaction between disease/phenotype associated SNPs and VDR binding. Furthermore, as it is clear that a SNP in linkage disequilibrium with a GWAS SNP may be contained within a VDR binding region and we also included these SNPs in the total list of SNPs examined (Fig. 1 Top). From these analyses, we applied transcription factor motif searching and exploited other ChIP-seq data to identify significant interactions between the VDR and other transcription factors, notably NF-κB. Taken together, we provide a statistically robust approach and strong analytical framework centered around transcription factor binding and the impact of genetic variation to infer functionally significant phenotypic consequences of genetic variation on transcription factor function.

## Results

### VDR binding overlaps with Lead and LD SNPs associated with immune phenotypes

The analyses of the individual data sets from five VDR ChIP-seq studies [8–11, 68] (Additional file 1: Table S1) yielded a VDR consensus cistrome consisting of 23,409 non-overlapping genomic binding sites [68]. Lead SNPs (10,432) were those downloaded directly from the GWAS catalog, and LD SNPs selection resulted in 181,050 SNPs, respectively, for a total of 191,482 SNPs associated with 930 unique phenotypes (as defined in the GWAS catalog) from 1,566 studies (Fig. 1 Bottom and Additional file 1: Table S2). The overlap of genomic coordinates of these 191,482 SNPs with the 23,409 VDR loci identified a total of 574 disease- or phenotype- associated SNPs representing 211 unique traits under 506 unique VDR loci (Fig. 1 Bottom and Additional file 1: Table S3). Hypergeometric analyses corrected for multiple testing yielded 42 unique SNPs associated with seven different phenotypes. That is, the 42 SNPs that are significantly associated, at the genome-wide level, with a disease- or phenotype-associated trait, are also significantly overrepresented at VDR binding loci in individual and/or the combined dataset (Table 1).

**Table 1:** List of trait SNPs showing significant enrichment for associated traits

Trait	(A) Trait SNPs (No of SNPs associated with the trait (GWAS + LD)	(B) No. of Trait SNPs under VDR peaks	LD SNP/Lead SNP	Total No. of SNPs under VDR peaks	Corrected *p*-value	VDR peak set
Esophageal cancer (squamous cell)	160	7	rs4686848/rs2239612 rs2097461/rs2239815 **rs2239815/rs2239815** rs2269577/rs2239815 rs5752809/rs2239815 rs5752812/rs2239815 rs763073/rs2239815	574	0.0001	Combined
	160	4	rs2097461/rs2239815 rs2269577/rs2239815 rs5752809/rs2239815 rs5752812/rs2239815	149	0.002	GM10855.D3
Leprosy	187	4	rs11231770/rs538147 rs11231771/rs538147 rs10897487/rs538147 rs475032/rs538147	78	0.0003	LS180.D3
Mean corpuscular hemoglobin	148	5	rs4366668/rs6494537 rs2974750/rs11085824 rs131807/rs470119 rs131806/rs470119 rs131804/rs470119	574	0.02	Combined
	148	4	rs4366668/rs6494537 rs2974750/rs11085824 rs131806/rs470119 rs131804/rs470119	121	0.0006	GM10861.Veh
	148	3	rs4366668/rs6494537 rs2974750/rs11085824 rs131806/rs470119	81	0.008	GM10855.Veh
Helicobacter pylori serologic status	22	3	rs10012017/rs10004195 rs10034903/rs10004195 **rs10004195/rs10004195**	574	0.008	Combined
	22	3	rs10012017/rs10004195 rs10034903/rs10004195 **rs10004195/rs10004195**	316	0.001	GM10861.D3
Vitiligo	463	8	rs10824732/rs11593576 **rs10876864/rs10876864** rs4807000/rs6510827 rs41271391/rs59374417 rs16829980/rs59374417 rs885654/rs59374417 rs867234/rs59374417 rs7628982/rs9851967	574	0.02	Combined
	463	7	rs10824732/rs11593576 **rs10876864/rs10876864** rs4807000/rs6510827 rs41271391/rs59374417 rs16829980/rs59374417 rs885654/rs59374417 rs867234/rs59374417	316	0.003	GM10861.D3
	463	5	**rs10876864/rs10876864** rs41271391/rs59374417 rs16829980/rs59374417 rs885654/rs59374417 rs867234/rs59374417	149	0.007	GM10855.D3
Self-reported allergy	400	6	**rs10174949/rs10174949** rs10178845/rs10174949 rs5743566/rs2101521 rs5743565/rs2101521 rs45588337/rs2101521 rs55830619/rs2101521	316	0.012	GM10861.D3
Celiac disease	576	9	rs1250567/rs1250552 rs1250568/rs1250552 rs12924957/rs12928822 **rs12928822/rs12928822** rs34289272/rs13098911 rs71327040/rs13098911 rs17035355/rs17035378 rs2984920/rs2816316 rs4310388/rs3748816	574	0.016	Combined

For each trait, the number of SNPs (GWAS + LD; (B)) under VDR peaks was compared to the total number of SNPs (GWAS + LD; (A)) associated with the trait. The significance of enrichment was tested by a hypergeometric test and the *p*-values were corrected using Bonferroni (corrected *p*-value). LD SNP/Lead SNP shows the SNPs under VDR peaks (Lead SNP and/or LD SNPs). The lead SNPs which are also present under VDR peaks are shown in bold. VDR peak set is the the number of data sets in which the VDR peak and SNP relationship was found; Combined = all the peaks from six studies considered together, or cell line and treatment specific data set (D3 = treated with VDR ligand_,_ Veh = vehicle control). Peaks represent the data sets in which the traits showed significant enrichment. This analysis revealed 42 unique SNPs associated with VDR binding and enrichment of a trait

To attempt to gauge if a VDR-SNP-trait relationship was tissue specific we considered how common the relationships were across the different ChIP-seq data sets. The seven traits significantly associated with the 42 disease- or phenotype-associated SNPs were identified in the lymphoblastoid cells and also in one other data set, except for leprosy, which showed significant enrichment in only the VDR ligand-stimulated colorectal adenocarcinoma LS180 cells. Esophageal cancer (squamous cell), showed significant enrichment in more than one VDR dataset (*p* = 0.0001), and it was also amongst the significant traits within the ligand-stimulated ChIP-seq dataset in the CEPH cell line. Five of the other seven traits identified that associated with VDR peaks had predominant immune phenotypes including *Helicobacter pylori* serologic status, self-reported allergy and Celiac disease (Table 1 and Additional file 1: Table S4).

### Phenotype associated SNPs identify regions where the VDR binding is coincident with other transcription factors

Previously, we mined the consensus VDR cistrome for canonical DR3 motif using the de novo motif prediction tool, HOMER [69]. Searching under a low stringency motif score setting 38% of these sites (8,897) contain canonical DR3 motifs [68] and 2.6% (*n* = 15) of the 574 disease- or phenotype-associated SNPs were under a VDR peak that contained a DR3 motif (Table 2). Of these 15 SNPs, rs10174949 (Lead) and rs16829980 (*r* ^2^ = 1 with another Lead SNP, rs59374417), were contained within the 42 SNPs reported above that survived multiple test correction.

**Table 2:** SNPs present in DR3-type motifs contained within VDR peaks and associated with traits

Chr	Chr Start	Chr End	Strand	Reference Allele	Observed Alleles	SNP under VDR peak (Lead in bold)	Derived From (Lead SNP ID)	Trait	DR3 HOMER score	DR3 motif sequence (Homer)	SNP position in the motif
chr2	8442247	8442248	+	G	A/G	***rs10174949*** ^**a**^	***rs10174949***	***Self-reported allergy***	***4***	AGATCATCGGGGTTT	11
chr15	50946814	50946815	+	A	A/G	rs11070802	rs2414059	QT interval	7	AGTGCACATGGTGCA	7
chr3	119278467	119278468	+	A	A/G	**rs16829980** ^**a**^	**rs59374417**	**Vitiligo**	6	AGTTCACTCAGTACT	5
chr19	49867015	49867016	+	C	C/G	rs2288480	rs2303759	Multiple sclerosis	4	GTGTCACCAAGGGCG	7
chr4	95088557	95088558	+	C	C/T	rs28613890	rs11097407	Bipolar disorder and schizophrenia	8	GTGGTCATTGAGTTC	14
chr6	109704441	109704442	+	T	C/T	rs3757230	rs1046943	Height	7	AGGTCATTGGGGAGG	3
chr6	109704450	109704451	+	A	A/C	rs3757231	rs1046943	Height	7	AGGTCATTGGGGAGG	12
chr5	95265555	95265556	+	G	A/G	rs4563648	rs7700895	IgG glycosylation	5	GGGTGAGAAAGTTTC	2
chr1	205641342	205641343	+	C	C/G	rs55915134	rs16856186	Pulmonary function decline	6	TGGGTGAGGGGGGGG	6
chr11	118575418	118575419	-	G	C/T	rs625735	rs494459	Height	9.18	GTGTCAAAGGGTTCA	10
chr11	128206409	128206410	+	C	C/T	*rs6590322*	*rs6590322*	*Hippocampal atrophy*	***8***	AAAGTCAGAGAGGAC	7
chr15	49167995	49167996	+	G	A/G	rs73402209	rs8023445	Major depressive disorder	9.18	AGGTCAAAGAGGTCG	14
chr21	35348499	35348500	-	C	A/G	rs743418	rs2032314	Red blood cell traits	4	AGGCTGCTGGGTCCA	12
chr5	150169882	150169883	+	A	A/T	rs74973123	rs13361189	Crohn’s disease	5	ATGTTAAAGGGTTTA	7
chr9	123664122	123664123	+	A	A/G	rs7859805	rs881375	Rheumatoid arthritis	6	GGGGCAAAAGGTGTG	5

All the 574 disease- or phenotype-associated SNPs were overlapped with 8,884 VDR ChIP-seq loci containing DR3 motifs to identify SNPs present in DR3 motif. This analysis identified 15 SNPs to be present in DR3-type motifs. Table shows the details of 15 SNPs including homer score, associated Lead SNPs and traits/phenotypes. SNPs in bold survive multiple test correction

SNPs in bold are LEAD SNPs

^a^SNPs common with 42 significant trait SNPs, bases in red in motif sequence show the position of the SNP in the motif

Given that 40 of the 42 significantly associated GWAS SNPs were not linked to a DR3 motif, we mined these regions for significant enrichment of other transcription factor associations. In the first instance we mined these regions for transcription factor binding using RegulomeDB [20]. Analysis of these 42 SNPs with RegulomeDB revealed that 39/42 SNPs were predicted to lie in a region bound co-incident with other transcription factors (Additional file 1: Table S5). Filtering these interactions to only consider SNPs that had a high RegulomeDB score revealed phenotype-associated SNPs that significantly impacted transcription factor binding (Table 3). Six had scores from 1b-1f (likely to affect binding and linked to expression of a gene target e-QTLs) and nine had scores from 2a-2b (likely to affect binding) (Table 3 and Additional file 1: Table S5). It is also interesting to note the breakdown of all NHGRI SNPs studied (Table 3). 7324/191482 SNPs (3.8%) are in transcription factor binding regions and are predicted to change binding significantly with a score of 1 or 2. This is increased to 37% when considering these SNPs under VDR peaks and remains at 35% when considering VDR peaks significantly associated with a trait. These findings further support that the concept that these SNPs are functionally relevant for the disruption of transcription by multimeric transcription factor complexes that contain the VDR.

**Table 3:** The distribution of Regulome DB scores for different SNP lists

Regulome DB score	(A)	(B)	(C)
	No. of GWAS + LD SNPs (191482)	No. of (A) SNPs under VDR peaks (572)	No. of (B) SNPs under VDR peaks enriched for trait association (42)
1a	21	1	0
1b	272	15	1
1c	6	0	0
1d	148	3	0
1e	7	0	0
1f	3182	43	5
2a	369	34	2
2b	3182	109	7
2c	137	8	0
Total	7324	213	15

All the GWAS + LD SNPs were analyzed using RegulomeDB. The table shows the distribution of scores 1 and 2 SNPs under VDR peaks as well as for 42 significant trait associated SNPs

**Fig. 2:**
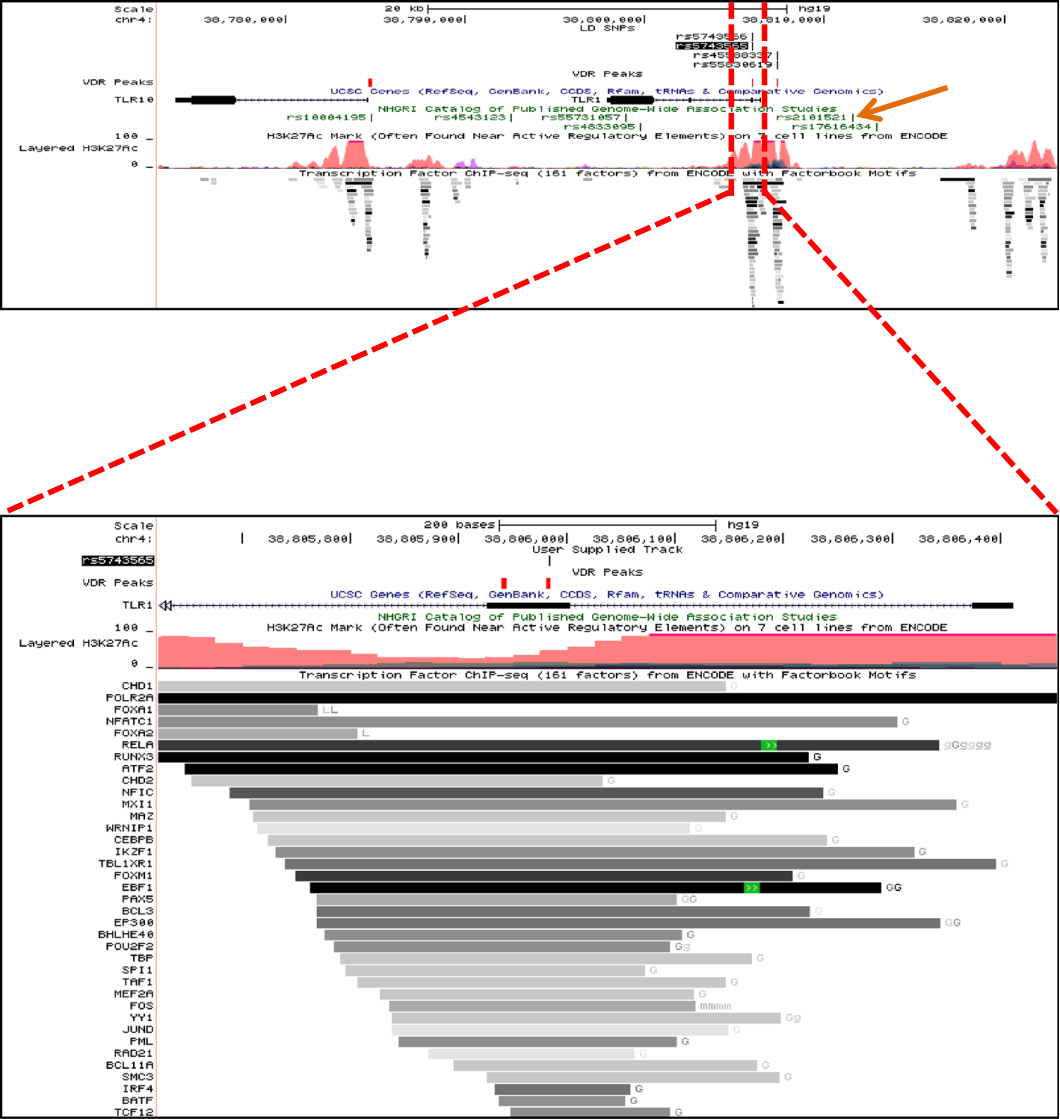
Function of a SNP contained within a VDR binding peak associated with Toll-like receptor 1 (TLR1). The *top panel* shows the presence of rs5743565 (*blocked out*), which is in LD with rs2101521 (*orange arrow*) associated with self-reported allergy in GWAS study. VDR peaks are shown as Red block custom tracts. The rs5743565 is associated with expression of the genes *TLR10, TLR6* and *TLR1*. The bottom panel shows the ENCODE ChIP-seq data for binding of different transcription factors in the region harboring rs5743565 and VDR binding sites (*in red*). The presence of rs5743565 in the binding region is predicted to alter the association of multiple transcription factors and the expression of associated genes, and therefore been assigned a score in RegulomeDB of < = 2

The diversity of the transcription factors identified suggest a high level of VDR-protein interactions that may represent VDR binding in an indirect or *trans* relationship with gene regulation. That is, the VDR is recruited to another protein complex, which is in turn in direct contact with DNA. Focusing on the 15 trait-associated SNPs that also had RegulomeDB scores of ≤2 suggest common VDR interactions with 95 different transcription factors that were co-enriched in binding sites for the VDR. For example, Fig. 2 illustrates the *TLR1* gene locus, and in particular the location of the rs5743565 SNP that is in LD with Lead SNP rs2101521, reported to be significantly associated with self-reported allergy [70]. This particular region of the gene also illustrates that it is commonly found to be associated with the open chromatin histone mark H3K27ac and is coincident with the significant binding of more than 30 different transcription factors as revealed by ENCODE investigators and reported in RegulomeDB.

The RegulomeDB approach is highly comprehensive approach and based on the actual binding of transcription factors as identified by ENCODE. We complemented this by mining the 42 SNPs with HaploReg [71] to identify the predicted impact of these SNPs on regulatory motifs. The predicted impact of the 42 disease- or phenotype-associated SNPs on the affinity of regulatory motifs is shown in Additional file 1: Table S6. Out of 42 SNPs analyzed, 37 SNPs showed potential to affect one or more motifs. The HaploReg analyses also showed that the 42 SNPs were highly enriched for strong enhancers and DNase hypersensitive regions across multiple cell lines (Additional file 1: Table S7).

Together the analyses of 42 disease- or phenotype-associated SNPs with RegulomeDB and HaploReg suggest that these SNPs affect binding motifs of important transcription factors. Also, they are present in enhancer and DNAse sites, suggestive of active transcription factor binding and they have a significant relationship with an altered gene regulatory capacity.

### Co-enrichment of VDR and NF-κB binding in CEPH cell lines

**Fig. 3:**
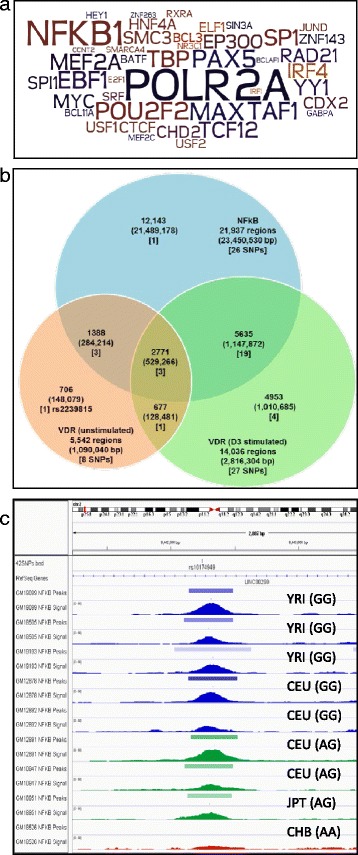
The co-occurrence of VDR and NF-κB1 binding at sites of significant genetic variation. **a** The wordcloud characterizes the transcription factors binding at the location of significantly associated Lead SNPs (and not linked to a DR3 motif) were identified by RegulomeDB; 95 different transcription factors, representing eight unique subgroups, overlapped with, and their function was altered by, these SNPs. The font size of transcription factor related to the number of times it was identified to associate with a significant SNP site. **b** The intersection of VDR and NF-κB ChIP-Seq from CEPH cell lines. The VDR ChIP-seq in the unstimulated and ligand-stimulated states in the CEPH cell lines GM10855 and GM10861 were intersected to generate a consensus cistrome for VDR binding sites in the unstimulated and stimulated states. Similarly, a consensus NF-κB cistrome was generated by intersecting the ChIP-Seq from the cell lines GM12878, GM12891, GM12892, GM15510, GM18505, GM18526, GM18951, GM19099, GM19193. These consensus cistromes were then intersected to reveal the unique and shared binding sites, and the overlap with the identified SNPs. **c** The effect of rs10174949 genotype on NF-κB binding in ChIP-seq from HapMap cell lines. The altered allele of rs10174949 was predicted to decrease the strength of predicted affinity of NF-κB1 by HaploReg. The ENCODE ChIP-seq data sets of NF-κB for HapMap cell lines for which genotype data was available were downloaded into Integrative Genomics Viewer (IGV). Population ID and the genotype for each cell lines is shown. Cell lines with homozygous reference allele are shown in *blue*, heterozygous samples are shown in *green* and sample with homozygous altered allele is shown in *red*. Samples with the homozygous altered allele (AA) completely lost NF-κB binding compared to the homozygous (GG) or heterozygous (AG) reference alleles

RegulomeDB analysis [72] of the transcription factors associated with all 42 SNPs established the most significantly impacted transcription factor binding. NF-κB was the most frequently enriched transcription factor when considering either all 42 SNPs or focusing on the 15 SNPs with RegulomeDB scores of 1 and 2. We chose to focus on the transcription factor enrichment amongst the 15 SNPs with high RegulomeDB scores as we reasoned these are likely to be functionally relevant. To illustrate the frequency, we have adopted a similar approach to motif analyses in which the size of the nucleotide letter is proportional to significance. In the current study we have exploited a wordcloud approach to visualize the level of significance of the transcription factors. Reflecting the active levels of transcription from these regions the RNA polymerase POLR2A is very commonly represented. However this does not bind in a motif-specific manner, rather it is recruited through protein-protein interactions. Clearly, after POLR2A, NFKB1 is prominently featured (Fig. 3a) as indicated by the font size of the transcription factor.

These findings suggested that disease and trait associated SNPs were significantly associated with VDR and other transcription factor, most prominently with NF-κB. To test how the common the co-occurrence of the VDR and NF-κB binding sites we exploited CEPH cell lines for which there are relevant ChIP-seq data. These cells were chosen as they are not transformed and therefore transcription factor binding is not likely to be altered. Specifically, we examined the genome-wide overlap between basal and 1α,25(OH)_2_D_3_-stimulated VDR and TNFα-stimulated NF-κB. The Venn diagram illustrates the overlap between these three cistromic data sets (Fig. 3b). Notably, the intersection between 1α,25(OH)_2_D_3_ stimulated VDR and TNFα stimulated NF-κB cistromes is pronounced, with 5,635 genomic regions shared. Furthermore, these shared binding regions also contained 22 of the 42 disease- or phenotype-associated SNPs (Fig. 3b). These findings further suggest the potential biological importance of the interaction between these two transcription factors. Finally, all of the SNPs reported to affect gene expression (e-QTL) were associated with immune phenotypes (Table 4).

**Table 4:** Showing e-QTLS and associated phenotypes for SNPs under VDR peak significantly enriched for trait association

Chrom	ChromStart	ChromEnd	Strand	Reference Allele	Observed Alleles	SNP under VDR peak (Lead + LD SNP)	RegulomeDB score	e-QTL (affected gene)	Associated GWAS Trait
chr4	38805982	38805983	-	T	A/G	rs5743565	1b	*TLR10/TLR6/TLR1*	Self-reported allergy
chr4	38784723	38784724	+	T	A/T	rs10004195	1f	*TLR6*	Helicobacter pylori serologic status
chr11	64140703	64140704	+	C	C/T	rs10897487	1f	*CCDC88/FLJ37970*	Leprosy
chr11	64140736	64140737	+	G	C/G	rs475032	1f	*CCDC88/FLJ37970*	Leprosy
chr12	56401084	56401085	+	G	A/G	rs10876864	1f	*RPS26*	Vitiligo

Five of 42 trait-associated SNPs which were e-QTLs with RegulomeDB score 1, e-QTL gene and traits associated with these SNPs in GWAS studies are shown

Figure 3c shows an example of how genetic variation may influence NF-κB binding. Specifically, rs10174949 on chromosome 2p lies in an intronic region of *LINC00299* and is reported to be significantly associated with self-reported allergy [70]. HaploReg revealed that the LOD score was 12 in the presence of reference allele, whereas the altered allele reduced the LOD score to 0.3. These findings suggest the altered allele (A) results in a loss of predicted binding strength compared to the reference allele (G). These predictions were supported by analyses of rs10174949 alleles in NF-κB ChIP-Seq data available on nine different CEPH cell lines from HapMap. Four out of five HapMap cell lines with the wild-type allele (GG) have a pronounced NF-κB binding peak at this region (Fig. 3c, peaks in blue), two of the three heterozygote cell lines had intermediary binding (peaks in green) whereas the one cell line with the homozygote (AA) genotype showed little or no binding (peak in red).

## Discussion

The goal of the current study was to test the hypothesis that the pleotropic actions of the VDR could be dissected by integrating VDR ChIP-Seq studies with GWAS findings in a robust statistical framework. The approach we describe is significant for several key reasons; the potential impact on the understanding of VDR biology, the generalizability of the approach to other nuclear hormone receptors and its potential for resonance with biological investigators.

Firstly, the impact of genetic variation on VDR function has been very intensively investigated almost exclusively in the context of SNPs either within the VDR gene itself or genes that encode proteins that in turn regulate VDR function (reviewed in [73–76]). Whilst these investigations highlight a role for VDR in human health and disease, they are ultimately limited in terms of scope of how genetic variation is considered and perhaps as a result, few of the findings have been replicated at the genome-wide level. By contrast, the current study identifies genetic variation that is significant at the genome-wide level is enriched in VDR binding sites and impacts VDR function. This finding informs understanding of the pleotropic actions of the VDR [44–60]. This has not been reported previously.

Secondly, the approach is applicable to other members of the nuclear hormone receptor superfamily, as well as other disease relevant transcription factors. Interestingly, despite their developmental and therapeutic relevance, nuclear hormone receptors are not comprehensively covered by either ENCODE or Roadmap Epigenome consortia. However, given the preeminent position of nuclear hormone receptors in human health and disease, other members of this superfamily have been extensively investigated by ChIP-seq approaches by other investigators. Indeed, there are hundreds of ChIP-seq datasets available for nuclear hormone receptors that have not been considered in the RegulomeDB approach that builds on ENCODE data. For example, the androgen receptor has been extensively investigated. This receptor is intimately associated with prostate cancer and other areas of men’s health [77, 78] and is associated with breast cancer in women [79, 80]. Multiple investigators have examined this receptor and to date there are over 130 individual ChIP-seq experiments for this receptor. Similarly, the estrogen receptor, which is intimately associated with breast cancer, bone health and a range of other phenotypes in women’s health [81, 82] has also been extensively investigated with over 200 ChIP-seq data sets available. Outside of the sex steroids, the glucocorticoid receptor is arguably one of the most prominent drug targets in human health being the central therapeutic target for a wide range of anti-inflammatory therapies [83] and its actions are central to wide spectrum of human phenotypes and diseases. Similarly, approximately 40 ChIP-seq datasets are currently available for this receptor. Similar analyses can be undertaken for other nuclear hormone receptors including peroxisome proliferator-activated receptors (PPARs) and the retinoic acid receptors (RARs) and can be extended to other clinically important transcription factors. Therefore the approach described in the current study has a significant potential to resonate with investigators who work with these other receptors.

We propose that these ChIP-seq datasets are very attractive to build consensus cistromes in different cell states for a given transcription factor. Specifically, the intersection of each of the different cistrome data sets established in different cell backgrounds can be generated to increase the statistical accuracy of the binding sites. Integration of these consensus cistromes with the NHGRI GWAS catalog has the strong potential to highlight how these receptors relate to human health and disease and specifically, what critical pathways are modulated by genetic variation. In this regard the current study is an important proof of principle that can be relatively quickly undertaken to highlight new avenues of biological function that can be exploited in diagnostic or therapeutic settings.

Finally, the current study has strong potential to resonate with biologists who study in depth either single members or related members from transcription factor superfamilies. These researchers bring considerable insight to research questions around a given transcription factor, but perhaps are less comfortable considering the intersection of multiple genome-wide data sets as a functional genomics approach. The current study therefore has the potential to serve as a guide for data integration and functional genomic discovery.

Applying this approach allowed us to be able to overlay a consensus VDR cistrome with data from the NHGRI GWAS catalog of SNPs, and identify disease and phenotype-associated variants that were genome-wide significant in the GWAS catalog and present at genomic VDR binding loci. Of these relationships, the most pronounced and significant related phenotype to emerge was associated with immune functions and capacity. What is also important is what did not emerge. Namely genetic variants associated with cancer risk were not commonly identified.

Within this framework, our study has demonstrated a number of intriguing findings. Using genome wide analyses of germline genetic variation and ChIP-seq data we identified the VDR binding loci significantly enriched for 42 disease- or phenotype-associated SNPs (significant GWAS findings and/or LD SNPs). We selected SNPs that were in LD, rather than in a certain genomic window (e.g., 10 kb from the binding site), as we wished to leverage understanding of genomic structure to inform how genetic variation impacted VDR function. Of these 42 SNPs, only two were associated with canonical DR3-type VDR binding sequences, with the majority of SNPs associated with other transcription factors, some of which are known to interact with the VDR. The analyses of shared enrichment of transcription factors by RegulomeDB and Haploreg also suggested that VDR interacts, perhaps in shared multimeric complexes with other transcription factors and potentially warrant further exploration given the shared platform of identification. The finding that the significantly enriched disease- or phenotype-associated SNPs are not commonly found in canonical DR3 VDR motifs is intriguing. All the published VDR ChIP-Seq identified significant enrichment of the DR3 motif but it was at best found in approximately 30% of VDR peaks [23]. However, in the current study, the number of sites with disease- or phenotype-associated SNPs did not appear as high as this, and arguably was even less common. These findings collectively suggest that the VDR may interact with the genome in a number of direct and indirect mechanisms. The indirect, *trans*, mechanisms remain enigmatic but the current findings suggest a functional interaction between VDR and NF-κB.

We have confidence in the strength of the findings. Analysis of the 42 disease- or phenotype-associated SNPs using ENCODE ChIP-Seq data demonstrated that 15 of these SNPS are “likely to affect transcription factor binding,” designated by a Regulome score ≤2. Therefore 33.3% of our final SNP set is likely to affect transcription factor binding. Across all GWAS and LD SNPs, ENCODE characterizes ~17% as likely to affect transcription factor binding. Therefore, a simple one-tailed Fisher’s exact test supports that indeed we can reject the idea that our SNP set is like that of the GWAS and LD background, meaning our SNPs show evidence of enrichment for affecting TF binding. Furthermore (as we have demonstrated) these functionally relevant SNPs are more frequently found when VDR appears to co-bind with another transcription factor explored, for example NF-κB.

Naturally, there are a number of caveats to these approaches and findings. For example, not all tissues express all the transcription factors highlighted by RegulomeDB and therefore interactions will be tissue specific. Similarly, another caveat is that DR3 binding elements may be under-represented in computational analyses due to technical reasons and incomplete understanding. However, it is worth stressing that each group that undertook the primary VDR ChIP-seq analyses reported that VDR regions containing DR3 elements represented a minority of VDR loci.

In a similar manner the significant enrichment SNPs associated with immune traits under VDR peaks in part reflects the role of VDR in immune function [84]. However these findings may reflect that VDR ChIP-seq were included from two lymphocyte CEPH cell lines. These cells contributed more VDR ChIP-peak enriched SNPs (442 SNPs) than either THP1 (43 SNPs), LS180 (80 SNPs) or LX2 (36 SNPs). Therefore we cannot exclude the possibility that this underpins the enrichment of immune phenotypes. Of course to counter this is the biological possibility that the VDR is intrinsically more genomically engaged in the GM cell lines, and hence the largest number of VDR peaks is identified in these cell models. Furthermore the GWAS catalog contains many more SNPs associated with non-immune phenotypes, for example cancer phenotypes, and these were not readily identified.

An intriguing new hypothesis generated by the current study is that genetic variants at the sites of VDR and NF-κB binding distort the control the of *Toll-like receptor 1 (TLR1)* and Long intergenic non- protein coding RNA 299 (*LINC00299*) to govern immunophenotypes. For example the genetic variants may change the on-off rates of the VDR- NF-κB complex binding at the site such that it alters the potential to regulate the downstream gene target. Identification of the sites of interaction in the current study actually make testing the significance of the sites through genome-editing approaches relatively easy for future studies (Figs. 2 and 3). In this manner it may be intriguing to edit the genome such that variants are added in and then the avidity of VDR and NF-κB can be tested with ChIP and Re-ChIP approaches as required. Such experiments would move towards understanding how genetic variation differentially impacts *cis* and *trans* binding events that in turn govern gene expression. Genome editing approaches may also be attractive for future studies as the SNPs in Table 2 are not highly penetrant, that is they are common variants with low/modest odds ratios, and are in syndromes that tend to be chronic and even adult onset. We believe that this reflects that VDR binding is part of a complex picture of transcription factor interactions that relate to disease. Thus looking for VDR binding at alleles associated with low/modest risk has a high probably of not demonstrating allele-VDR binding associations, not because the in silico experiments were incorrect but rather because these are complex disease phenotypes with several factors at play. Again, although the literature supports cooperation between these VDR and NF-κB signaling pathways, the current approach provides statistical significance to this interaction, and justifies the significant approaches required to test this in relevant biological settings. Precise genome editing approaches will allow the definitive testing of these possibilities.

The potential interactions between VDR and NF-κB are given further relevance as the principal disease phenotypes associated with the SNPs are enriched for immune-related phenotypes (Table 1). For example, leprosy and vitiligo have different pathophysiology but both have major immune components. Vitiligo is an autoimmune disease [85, 86], whereas defects in cell-mediated immunity have been reported in leprosy [87, 88]. Additionally, studies have shown the interaction of Toll-like receptors (TLRs) and the vitamin D anti-microbial pathway contribute to leprosy outcomes [88]. Given the large number of individuals around the world who are currently enrolled in vitamin D intervention and supplementation trials [41, 42, 89–96] the current findings add to the genomic framework for interpreting the health responses, and for investigating the genetic basis for sensitivity and resistance to vitamin D exposure and VDR function. A further interesting finding of the current study is to some extent in the negative findings. Despite the large body of literature supporting roles for the VDR in various cancers (reviewed in [24]), only one phenotype associated SNP related to cancer and this suggests that genetic variation does not impact VDR signaling in cancer phenotypes to a significant extent.

## Conclusion

Taken together the current study has examined VDR transcriptome behavior through integrative genomic approaches and demonstrated a number of intriguing and statistically robust findings. Firstly, that the VDR binding peaks were significantly enriched for 42 diseases or trait associated SNPs, identified through GWAS studies. Secondly that only two of these SNPs were associated with canonical DR-3 type VDR binding elements and the majority of SNPs were associated with other transcription factors some of which are known to interact with the VDR, whereas others are novel. Finally the disease phenotypes are overwhelmingly enriched for immune-related phenotypes. Given the large number of individuals around the world who are currently enrolled in vitamin D intervention and supplementation trials, the current findings provide a framework for interpreting the health responses and for investigating genetic the basis for sensitivity and resistance to VDR function.

## Methods

### Study design

The study design is summarized in Fig. 1 and Additional file 1: Table S2. Briefly, we merged peaks identified in multiple VDR ChIP-seq data sets to build a consensus VDR cistrome which was then overlapped with both SNPs identified by GWAS (we call the SNPs that are reported to be statistically associated with a phenotype lead SNPs) and SNPs in LD with lead SNPs. We then identified lead plus LD SNPs under VDR peaks and Hypergeometric testing was used to identify phenotypes enriched for presence under VDR peaks. SNPs were further annotated based on presence of absence of a VDR motif e.g., a motif was identified as present under peak or not. Statistically significant lead and LD SNPs were further annotated for function and interaction with other transcription factors.

### Statistical and bioinformatics analyses

#### VDR ChIP-seq data sets and peak identification

As the individual ChIP-Seq data sets had been analyzed using different workflows, we chose to re-align the reads, and define enriched peaks with the same harmonized workflow. In order to allow direct comparison across all the published VDR ChIP-seq data, each VDR ChIP-seq data set was analyzed again [68]. In summary, VDR ChIP-seq data from five studies (six data sets) were selected for analysis, including GM10855 and GM10861 lymphoblastoid cells [9], THP-1 monocyte-like cells [8], LS180 colorectal cancer cells [11], LX2 hepatic stellate cells [10] and LPS-differentiated THP-1 cells [68]. Sequence reads were aligned hg19 Bowtie software version 1.0.0 [97] and significant peaks were identified using MACS version 2 [98] with the following essential command line arguments: macs2 callpeak –bw 150 –keep-dup 1 –g hs —qvalue = 0.01 –m 5 50 —bdg. Otherwise, default parameters were used. From these the analyses the combined data set was generated which contained in total 23,409 non-overlapping VDR peaks across these samples and analyzed using HOMER to identify DR3-type sequences [68]. Briefly, each data set was searched for de novo motifs [99] on ±100 bp peak summit regions using the following essential command line arguments: annotatePeaks.pl < sample_name > hg19 –noann –nogene –m < motif_file > −size −100, 100. Detailed methods for this analysis are provided in our previous publication [68].

#### Selection of lead SNPs and linkage disequilibrium SNPs

To create a comprehensive SNP list associated with disease/phenotypes at genome-wide significance level we downloaded the NHGRI GWAS Catalog (dated: 12/15/2015) [100]. We used SNP Annotation and Proxy Search (SNAP) tool to identify SNPs in strong linkage disequilibrium (LD) with these GWA SNPs we used HapMap and 1000 genome data from the Centre d’Etude du Polymorphisme Humain (CEU) population [101]. LD (r^2^) and minor allele frequency (MAF) thresholds for SNP selection were 0.8 and 0.05, respectively, considering all SNPs within a 500 kb region surrounding the Lead SNP. This yielded a final list of significant SNPs from replicated GWAS plus LD SNPs (S_N_).

#### Identification of statistically significant SNPs in VDR binding regions for each GWA phenotype

We used a hypergeometric test to assess an over or under representation of GWAS and LD SNPs under peaks for each trait/phenotype and to determine if SNPs associated with a particular phenotype were more frequently under a VDR Chip-seq peak than expected. All analyses were done in R using GenomicRanges [95]. To test for evidence of statistically significant enrichment of SNPs associated with a particular trait/phenotype under VDR peak we used a hypergeometric test using phyper function in R and applied bonferroni correction for the number of phenotypes tested. In addition to S_N_ and S_P_ for each trait we identified the total number of both GWAS and LD SNPs (S_n_) and then how many of those SNPs overlap with VDR peaks (S_pi_ for all i = 1 to 211, where i reflects each phenotype of the 211 phenotypes tested). This information was then used to perform the hypergeometric test using phyper function in R for each trait. A Bonferroni correction was used to control for the number of phenotypes tested.

#### Functional annotation of significant SNPs

To understand the function consequences of the SNPs showing evidence of enrichment under VDR peaks we used two different in silico methods with a specific focus on chromatin status around the SNPs and how the SNPs affect binding motifs of different transcription factors. First, we utilized RegulomeDB [20] to identify important chromatin signatures and transcription factors binding to the SNPs. RegulomeDB utilized ChIP-seq data for histone marks and transcription factors across multiple cell lines from ENCODE [1, 102]. Based on these data sets RegulomeDB scores each SNPs for their function importance. SNPs with scores of 1a-f, 2a, 2b and 2c are thought to likely affect transcription factor binding. In addition we used HaploReg v2 to determine if the presence of each SNP affects the binding motif of a transcription factors based on position weight matrix [71]. HaploReg constructs position weight matrix for both reference and altered allele for each SNP from multiple data sources and provides a log-odds (LOD) score. The change in log-odds (LOD) score as alleles change (LOD_(altered allele)_ – LOD _(reference allele)_) was determined and as was the number of affected motifs. A negative LOD difference value indicates the predicted relative affinity is higher for the reference sequence whereas a positive value means that the predicted relative affinity is higher for the alternate allele.

#### Intersection of VDR and NF-κB binding sites in CEPH cell lines

We focused on the potential of VDR binding site-associated SNPs to impact NF-κB binding by examining their co-incident binding. To test the possibility of significant shared genomic binding VDR and NF-κB we established a consensus cistrome for each factor and then intersected these data sets. Specifically, basal and 1α,25(OH)_2_D_3_-stimulated VDR cistromes was generated by intersecting the ChIP-Seq from GM10855 and GM10861 cells. TNFα-stimulated NF-κB cistrome was generated by intersecting the ChIP-Seq from the cell lines GM12878, GM12891, GM12892, GM15510, GM18505, GM18526, GM18951, GM19099, GM19193. Overlaps of the different datasets were examined using GenomicRanges in R and were deemed positive if at least 25% of the peak genomic region overlapped.

## Additional files

Additional file 1: Table S1. Showing number of VDR peaks and number of Lead and LD SNPs overlapping with VDR peaks in different cell line models. Number of peaks for individual VDR ChIP-seq data and the number of Lead SNPs along with associated LD SNPs which fall under VDR peaks for each data set is shown here. **Table S2.** Study design, analytical methods and results. Summary table describing different stages of the analyses including the principal methods (computational approaches, databases and webtools) and key finding from each stage. **Table S3.** List of 574 SNPs (GWAS + LD SNPs) under VDR peaks. **Table S4.** List of 42 SNPs showing significant enrichment for associated traits. **Table S5.** RegulomeDB scores for 42 significant trait associated SNPs. **Table S6.** Summary of HaploReg results for 42 trait associated SNPs. **Table S7.** HaploReg summary table of enrichment analysis of enhancers and DNase sensitive regions associated with 42 trait associated SNPs. (XLSX 94 kb)

## Abbreviations

CEPH: Centre de’Etude du Polymorphism Humain
CTCF: CCCTC-Binding Factor
DR3: Direct repeat spaced by 3 bp
ENCODE: Encylopedia of DNA elements
eQTL: Expression quantitative trait loci
FOXO4: Forkhead Box O4
GATA4: Gata-Binding Protein 4
GWAS: Genome wide association studies
HNF4α: Hepatocyte Nuclear Factor 4-Alpha
HOMER: Hypergeometric optimization of motif enrichment
HSF1: Heat-Shock Transcription Factor 1
IGV: Integrative Genomics Viewer
LOD score: logarithm (base 10) of odds score
NFKB1: Nuclear Factor Of Kappa Light Polypeptide Gene Enhancer In B-Cells 1
NF-κB: Nuclear Factor Kappa-B
NHGRI: National Human Genome Research Institute
POLR2A: Polymerase (RNA) II (DNA Directed) Polypeptide A, 220 kDa
SMARCC1: Swi/Snf-Related, Matrix-Associated, Actin-DependentRegulator Of Chromatin, Subfamily C, Member 1
SP1: Sp1 transcription factor
STAT3: Signal transducer and activator of transcription 3
TSS: Transcription start site
VDR: Vitamin D receptor
